# Shexiang Tongxin Dropping Pills targeting Piezo1/Ca^2+^/NLRP3 axis attenuates vascular endothelial inflammation

**DOI:** 10.1186/s13020-026-01402-3

**Published:** 2026-04-29

**Authors:** Jingya Liu, Ke Wang, Hongjing You, Liang Long, Yue Xu, Qiuhe Chen, Yang Chen

**Affiliations:** 1 Chinese Medicine Guangdong Laboratory, Zhuhai, 519031 Guangdong China; 2 State Key Laboratory of Traditional Chinese Medicine Syndrome, Key Laboratory of Chronic Disease Prevention and Control of Traditional Chinese Medicine of Guangdong Higher Education Institutes, School of Pharmaceutical Sciences, Guangzhou University of Chinese Medicine, Guangzhou, 510006 Guangdong China; 3 State Key Laboratory of Traditional Chinese Medicine Syndrome, Key Laboratory of Chronic Disease Prevention and Control of Traditional Chinese Medicine of Guangdong Higher Education Institutes, Science and Technology Innovation Center, Guangzhou University of Chinese Medicine, Guangzhou, 510006 Guangdong China; 4 China Science and Technology, Development Center for Chinese Medicine, Beijing, 100027 China

**Keywords:** STDP, Vasculitis, Vascular endothelial inflammation, Piezo1, NLRP3 inflammasome

## Abstract

**Background:**

Vascular endothelial inflammation is a key pathological process underlying various inflammatory vascular conditions, which can lead to severe cardiovascular complications. Shexiang Tongxin Dropping Pills (STDP) is a traditional Chinese medicine formulation clinically used for cardiovascular disorders and has shown protective effects on the vascular endothelium. However, the specific mechanisms by which STDP attenuates vascular endothelial inflammation remain incompletely elucidated.

**Methods:**

An experimental model of LCWE-induced vasculitis was established by intraperitoneal injection of lactobacillus casei cell wall extract (LCWE) in C57BL/6 mice. The effects of STDP were evaluated through whole blood analysis, EVG staining, and immunofluorescent staining for adhesion molecules (ICAM-1, VCAM-1) and macrophage infiltration. An in vitro endothelial damage model was established by stimulating murine vascular endothelial cells (MVECs) with LCWE. Network pharmacology, calcium imaging, and siRNA-mediated knockdown of Piezo1 were employed to elucidate the underlying mechanism.

**Results:**

STDP greatly alleviated the weight loss and reduced spleen coefficient in LCWE-induced mice. It decreased the proportion of monocytes in the blood, reduced the area and maximum diameter of the abdominal aorta, and preserved the structural integrity of elastic fibers in the abdominal aorta wall as revealed by EVG staining. Besides, STDP significantly alleviated vascular endothelial inflammation both in the mouse model and in the cell model, as manifested by inhibition of intercellular adhesion molecule-1 (ICAM-1) and vascular cell adhesion molecule-1 (VCAM-1) expression. Mechanistically, STDP significantly inhibited Piezo1 channel activation, thereby reducing calcium ion (Ca^2+^) influx. This inhibitory effect prevented the activation of the NLRP3 inflammasome in endothelial cells, thus protecting endothelial cells and alleviating inflammation.

**Conclusion:**

STDP attenuates vascular inflammation by downregulating Piezo1 expression, thereby reducing Ca^2+^ influx and suppressing NLRP3 inflammasome activation. The Piezo1/Ca^2+^/NLRP3 pathway may represent a novel therapeutic target for ameliorating vascular endothelial inflammation.

## Introduction

Vasculitis is a group of disorders characterized by the infiltration of inflammatory cells in the vascular wall and perivascular area, resulting in vascular damage, including fibrin deposition, collagen fiber degeneration, and endothelial cell and myocyte necrosis [1]. Vasculitis can lead to vascular stenosis, thrombotic occlusion, aneurysms, dissection, and even full-thickness rupture of the arterial wall. In addition to vascular inflammation, vasculitis may also affect the cardiovascular system by inducing accelerated atherosclerosis, hypertension, and thromboembolic events, thereby potentially leading to life-threatening diseases such as myocardial infarction and stroke [2]. Regrettably, despite significant advancements in vasculitis research over the past thirty years, its pathogenesis remains inadequately comprehended. Given the significant heterogeneity in the clinical manifestations of vasculitis and the lack of reliable diagnostic criteria [3], coupled with racial and regional variations in its incidence and symptoms [3, 4], considerable challenges exist in the timely diagnosis and standardized treatment of this disease in clinical practice. Collectively, these difficulties contribute to the refractory nature of vasculitis [5, 6]. Currently, the standard therapeutic strategy for vasculitis remains centered on glucocorticoids in combination with conventional immunosuppressive agents (e.g., cyclophosphamide, methotrexate) or biologics (e.g., rituximab, tocilizumab) [7]. However, long-term use of these agents is frequently associated with severe adverse effects, including increased risk of infections, metabolic disturbances, bone marrow suppression, and gonadal toxicity. Moreover, approximately 30%-50% of patients experience relapse upon dose reduction or discontinuation of therapy, and some patients are refractory to initial treatment, ultimately leading to irreversible organ damage [7, 8]. Therefore, exploring novel therapeutic strategies that are both effective and safe, and that can synergistically suppress inflammation while repairing the vascular endothelium, has become an urgent scientific priority in the field of vasculitis. Given that vascular endothelial inflammation is the central pathological process underlying vasculitis, understanding its molecular mechanisms may inform future therapeutic strategies. The present study focuses on this process using an experimental model of vasculitis induced by LCWE.

Vascular endothelial cells form the primary barrier of the vascular wall. They not only maintain vascular integrity and stability but also participate in regulating vascular tone, blood coagulation, and inflammatory responses, playing an irreplaceable role in the occurrence and development of cardiovascular diseases [9, 10]. Once endothelial dysfunction occurs, the expression of pro-inflammatory cytokines, chemokines, enzymes, and adhesion molecules is significantly up-regulated [11], and ion channels are opened. This disrupts ion homeostasis within the endothelium (e.g., Ca^2^⁺ [12, 13] and Na⁺ [14, 15]), leading to inflammasome activation and a range of vascular disorders [16]. Piezo1 is a mechanosensitive cation channel activated by various mechanical forces; it is widely distributed throughout the cardiovascular system and plays a crucial role in the development of cardiovascular diseases [17, 18]. Excessive opening of the Piezo1 channel leads to Ca^2^⁺ influx, ultimately resulting in cellular excitation and signal transmission. Notably, Ca^2^⁺ signaling is also a crucial factor for the activation of the NLRP3 inflammasome, which is the most classic and widely studied type of inflammasome implicated in inflammatory endothelial injury [9, 18, 19]. Upon activation, the NLRP3 inflammasome triggers the cleavage and activation of Caspase-1, which in turn mediates the cleavage and release of the pro-inflammatory cytokine IL-1β. This process leads to continuous damage to vascular endothelial cells [20]. Despite a clear understanding of the correlation between vascular inflammation and endothelial injury, a research gap remains regarding the specific mechanism by which vascular inflammation affects the opening of endothelial ion channels and the interaction between this process and NLRP3 inflammasome activation. Accordingly, alleviating vascular endothelial inflammatory injury represents a critical strategy for improving vascular function and preventing cardiovascular complications associated with inflammatory vascular injury.

Shexiang Tongxin Dropping Pills (STDP) are formulated from the ancient recipe “Zhi Baodan” from the Song Dynasty’s “Tai Ping Hui Min He Yao Gui Fang”. The formulation comprises artificial Moschus, total ginsenosides from the stems and leaves of Panax ginseng C.A.Mey. (verified with http://www.theplantlist.org, accessed on [May 03, 2025]), Bufo gargarizans Cantor venom, Salvia miltiorrhiza Bunge (verified with http://www.theplantlist.org, accessed on [May 03, 2025]), Bovis Calculus Artifactus, synthetic Borneolum, and Fel Ursi [21]. In both fundamental and clinical research, STDP has demonstrated advantageous impacts in the management of cardiovascular conditions [22]. Recent studies have demonstrated that STDP can safeguard vascular endothelial cells and limit the infiltration of inflammatory cells, as well as endothelial dysfunction [23, 24], reducing cardiomyocyte fibrosis. These effects suggest that STDP can protect vascular endothelial cells and limit inflammatory infiltration. Accordingly, we hypothesized that STDP might attenuate vascular endothelial inflammation, potentially through modulation of endothelial ion channels and inflammasome pathways.

Based on these considerations, we hypothesized that STDP may attenuate vascular endothelial inflammation by modulating Piezo1-mediated Ca^2^⁺ influx and thereby suppressing NLRP3 inflammasome activation. Therefore, this study aims to investigate whether STDP can alleviate vascular endothelial inflammation via the Piezo1/Ca^2+^/NLRP3 signaling pathway in an experimental model of LCWE-induced vasculitis.

## Material and methods

### Drugs

STDPs (National medical products number: Z20080018, product batch number: 221205) were provided by the Conba Pharmaceutical Co., Ltd. (Shanghai, China). STDP was dissolved in 1% sodium carboxymethyl. The high dose was set at 100 mg/kg/day, the medium dose at 50 mg/kg/day, and the low dose at 25 mg/kg/day. Aspirin (100 mg/kg/day) served as the positive control. STDP and Aspirin were intragastrically administered for a total of seven days and blood was taken from the orbital venous plexus at day 0 and 3 for whole blood analysis and humanely executed and sampled after 7 days.

### Quality control

STDP is a clinically approved and marketed traditional Chinese medicine with standardized production procedures and strict quality control. STDP was prepared according to established methodologies [21] in full compliance with National Medical Products Administration (NMPA) guidelines. Comprehensive quality control specifications and chemical fingerprinting data are documented in another study [25]. The stock solution of STDP was prepared according to previously reported methods. STDP powder was dissolved in sterile phosphate-buffered saline (PBS) containing 0.1% DMSO, vortexed thoroughly to prepare a stock solution. The stock solution was then filtered through a 0.22 μm filter and stored at -20 °C until use. For cell treatments, the stock solution was diluted with complete culture medium to the indicated working concentrations. Strict quality control was performed to ensure the stability, consistency, and reliability of the drug used in this study. The concentration and administration protocol of STDP were strictly referenced from previous peer-reviewed studies that have verified its effects on cultured endothelial cells [23, 26–28].

### Reagents and antibodies

STDP (Drug Administration Code: Z0080018). LCWE (Lactobacillus casei, ATCC, 11,578) extraction method adapted appropriately with reference to (Nourshargh and Alon, 2014). Aspirin (Asp, > 98% purity, TCI Development Ltd.) purchased in Shanghai, China. MCC950 (CP-456773, Med Chem Express, USA), Yoda1 (Sigma-Aldrich, SML1558, USA), Fura-2/AM (F1201, Invitrogen, USA), Lipo8000 (C0533, Beyotime, China), BAPTA-AM (HY-100545, MCE, China). Donkey serum (JAC-017-000-121, Jackson, USA). Antibodies used include: Anti-Piezo1 (DF12083, Affinity, Changzhou, China), ICAM-1 (sc-107, Santa, USA)、VCAM-1 (sc-13160, Santa, USA), NLRP3 (AF4620, Affinity, China), NLRP3 (45889, Novus, China), mature-IL-1β (WL00891, Wanlei, China), F4/80(A18637, ABclonal, China), Pro-IL-1β (WL02257, Wanlei, China), Caspase-1 (PA5-87536, Invitrogen, USA), GAPDH (60004-1-1G, Proteintech, China), Anti-mouse (7076 s, CST, USA), Anti-rabbit (7074 s, CST, USA), Donkey-anti-Sheep 555 (A21436, Invitrogen, USA), Donkey-anti-Rabbit 488 (A21206, Invitrogen, USA), Donkey-anti-mouse 488 (A21202, Invitrogen, USA), Donkey-anti-mouse 555 (A31570, Invitrogen, USA), Collagen fiber and elastic fiber staining kit (G1597, Solarbio, China), ELISA kits (IL-18: SEKM-0019, IL-1β: SEKM-0002, Solarbio, China).

### Animal experiments

The animal experiments in this study followed the guidelines of the National Institutes of Health (NIH) and were approved by the Institutional Animal Care and Use Committee of Guangzhou University of Chinese Medicine (approval number: ZYD-2023-091), and the experimental design was in accordance with the “Regulations on Ethical Review of Animal Experiments of Guangzhou University of Traditional Chinese Medicine”. Mice were housed in a temperature-controlled environment (22 ± 2 °C) with a 12-h light/dark cycle and had free access to standard laboratory chow and water. Male C57BL/6 J mice (3–4 weeks old, approximately 12 g) were purchased from Vital River Laboratories (Beijing, China), and were randomly divided into Control group, LCWE group, STDP low-dose group, STDP medium-dose group and STDP high-dose group and Aspirin 100 mg/kg positive control group. LCWE was injected intraperitoneally at a dose of 500 μg per mice [27], while the control group was injected intraperitoneally with 500 μL of PBS. Considering the young age of the mice, the administration of the drugs was initiated one day after the injection of LCWE. At the end of the study, mice were euthanized by intraperitoneal injection of pentobarbital sodium followed by cervical dislocation to ensure death, in accordance with institutional animal welfare guidelines.

### Whole blood analysis

Extract fresh mouse blood and place it in an EP tube containing an anticoagulant (Regan, R1012). Gently mix to prevent blood from clotting. Use a hematology counter (Mindray, BC2800 vet) to count the cells and record the complete blood analysis data.

### Cell culture

MVECs (murine vascular endothelial cells) were purchased from ATCC (CRL-2586, Shanghai, China) and cultured in a thermostat incubator at 37 °C with 5% carbon dioxide concentration using high glucose medium containing 10% fetal bovine serum (Gibico, USA). The MVECs were divided into six groups of plates: control group, LCWE model group (15 μg/mL), LCWE plus low, medium and high concentration of STDP administration group (0.125 μg/mL, 0.25 μg/mL, 0.5 μg/mL) [23, 26–28], and LCWE plus positive control aspirin group (2 mmol/L), with the number of cells in each group being administered at 2.0 × 10^5^ cells per well proteins were extracted after 24 h.

MVECs were loaded with 10 μM BAPTA-AM (in DMSO) in serum-free medium for 30 min at 37 °C, washed, and recovered for 30 min. Cells were then stimulated with LCWE (15 μg/mL) for 24 h. Controls received equivalent DMSO (< 0.1%).

### EVG staining

Mouse abdominal aortic tissues were collected, fixed in 4% paraformaldehyde, and embedded in paraffin. The specimens were sectioned at a thickness of 4–5 μm. Following deparaffinization and rehydration, the sections were stained using a collagen fiber and elastic fiber staining kit (G1597, Solarbio, China) according to the manufacturer's instructions. Briefly, sections were incubated in Verhoeff staining solution for 15–30 min, rinsed with tap water, and then differentiated in ferric chloride solution under microscopic observation until elastic fibers appeared purple-black against a gray-white background. After thorough washing, the sections were counterstained with modified EVG staining solution for 1–2 min, quickly rinsed with water, dehydrated through graded ethanol, cleared in xylene, and mounted with neutral resin. The staining results showed that elastic fibers were stained purple-black, collagen fibers were stained red, and muscle tissues were stained yellow.

### Immunofluorescence staining assay

For immunofluorescence staining of cardiac vessels, mouse heart cryosections (6 μm) were fixed in pre-cooled acetone, permeabilized with 0.3% Triton X-100, blocked with donkey serum, and incubated overnight at 4 °C with primary antibodies against vWF (1:400) combined with F4/80 (1:200), ICAM-1 (1:200), or VCAM-1 (1:200). After washing, sections were incubated with fluorescently labeled secondary antibodies (donkey anti-sheep 1:200, donkey anti-mouse 1:200) for 2 h at room temperature in the dark, then washed and mounted.

For cellular immunofluorescence, MVECs cultured on confocal dishes were fixed with 4% PFA, permeabilized, blocked, and incubated overnight at 4 °C with primary antibodies against VCAM-1 (1:200), NLRP3 (1:200), or Caspase-1 (1:200). Cells were then washed, incubated with corresponding fluorescent secondary antibodies for 2 h at room temperature, followed by DAPI staining. After final washes, images were captured using a confocal microscope.

For colocalization analysis, confocal images were processed using FIJI ImageJ software. Background subtraction was performed using the “Subtract Background” function. To ensure reliability of the results, negative control staining (sections incubated with secondary antibody only) was performed to identify and subtract non-specific autofluorescence, particularly from vascular elastic fibers and other tissue components. Manders overlap coefficients (M1 and M2) were calculated to quantify the fraction of overlap between the two fluorescent signals, with values ranging from 0 (no overlap) to 1 (complete overlap)[29]. For each sample, the MOC value of the negative control was subtracted. All analyses were performed by an investigator blinded to experimental grouping. At least five independent biological replicates per group were analyzed.

### Western blotting

Protein samples were separated by 8% or 10% SDS-PAGE, transferred to membranes, and blocked with 5% skimmed milk for 2 h at room temperature. Membranes were incubated overnight at 4 °C with primary antibodies against. After washing with TBST, membranes were incubated with HRP-conjugated secondary antibodies for 2 h at room temperature. Protein bands were visualized using enhanced chemiluminescence. The gray values of protein bands in Western blot experiments were quantified using ImageJ software (National Institutes of Health, USA). The expression level of each target protein was first calculated as the ratio of its band gray value to that of the corresponding internal reference (e.g., GAPDH or β-actin), and then normalized to the mean ratio of the control group, with the final results presented as relative expression levels. All experiments were independently repeated at least three times (N ≥ 3). Data are expressed as mean ± standard error of the mean (SEM). Statistical analysis was performed using GraphPad Prism 8.0 software. For multiple group comparisons, one-way analysis of variance (ANOVA) was used, followed by Tukey’s multiple comparison test. A *P*-value < 0.05 was considered statistically significant.

### Ca^2+^ imaging experiments

MVECs were divided into control group, LCWE group and LCWE + STDP groups, 24 h after administration, Hanks buffer salt solution (HBSS) was rinsed to wash off the medium and Fura-2/AM was incubated for 30 min at 37 °C protected from light. And HBSS was rinsed to ensure that excess Fura-2/AM was removed. Then HBSS was added and incubated at 37 °C protected from light for 30 min. Then add HBSS and incubate for 30 min at 37 °C, avoiding light. Observe in Metafloor software (Molecular Devices), after the baseline is stable, add Yoda 1, the final concentration is 5 μM [30, 31], and the instrument automatically recorded the fluorescence emission wavelength every 10 s. The change in intracellular Ca^2+^ concentration was calculated based on the ratio of the emission intensity of Fura-2 AM at 510 nm after excitation at 340 and 380 nm.

### Cell transfection

The silencing of Piezo1 in MVECs was performed using siRNA. MVECs were seeded in 6-well plates and grown to 60–70% confluence. All primers used in this study were designed and commercially synthesized by Tsingke Biotechnology Co., Ltd. (Beijing, China). The siPiezo1 or a negative control siRNA was transfected into the cells using Lipofectamine 3000 according to the manufacturer's instructions. The specific sequences for siPiezo1 were: Sense, 5′-CGACGAUGAAGACAUAGAU-3′ and Antisense, 5′-AUCUAUGUCUUUCAUCGUCG-3′. After 24 h of transfection, the silencing efficiency was validated by Western Blot before proceeding to subsequent experiments.

### Measurement of cell viability

MVECs cells were seeded in 96-well plates at a density of 6 × 10^3^ cells per well and cultured for 24 h. The cells were divided into five groups: control, LCWE, LCWE + 0.05 μg/mL STDP, LCWE + 0.1 μg/mL STDP, LCWE + 0.125 μg/mL STDP, LCWE + 0.25 μg/mL STDP, and LCWE + 0.5 μg/mL STDP. The effect of STDP on cell viability was assessed using the Cell Counting Kit-8 (CCK-8) assay (CA1211, Solarbio, China) according to the manufacturer's instructions.

### Measurement of lactate dehydrogenase (LDH) release

MVECs cells were seeded in 96-well plates at a density of 6 × 10^3^ cells per well and cultured for 24 h. The grouping was consistent with that described for the cell viability assay. The levels of LDH released into the culture medium were measured using an LDH assay kit (A020-2–2, Nanjing Jiancheng, China) following the manufacturer's protocol.

### ELISA

The grouping was consistent with that described for the cell viability assay. Following the respective treatments, cell culture supernatants were collected. The levels of interleukin-18 (IL-18) and interleukin-1β (IL-1β) were measured using commercial ELISA kits (IL-18: SEKM-0019; IL-1β: SEKM-0002, Solarbio, China) according to the manufacturer's instructions.

### Network pharmacology analysis

To investigate the potential mechanisms of Shexiang Tongxin Dropping Pills (STDP) in the treatment of vasculitis, a network pharmacology approach was employed. The active components of STDP and their corresponding targets were retrieved from the BATMAN-TCM database (http://bionet.ncpsb.org.cn/batman-tcm/) by searching its seven constituent herbs: Moschus, Bufonis Venenum, Fel Ursi, Salviae Miltiorrhizae Radix et Rhizoma, Bovis Calculus Artifactus, Borneolum Syntheticum, and Ginseng Folium, with a score cutoff of 20 (*P* < 0.05). All active components and their corresponding target proteins were collected, duplicates were removed. Vasculitis-related targets were obtained from the GeneCards database (https://www.genecards.org/) using the keyword “Vasculitis”. The obtained disease targets and STDP drug targets were imported into Venny 2.1.0 (https://bioinfogp.cnb.csic.es/tools/venny/) to identify overlapping targets representing potential therapeutic targets of STDP against vasculitis, and a Venn diagram was generated. Gene Ontology (GO) functional enrichment analysis and Kyoto Encyclopedia of Genes and Genomes (KEGG) pathway enrichment analysis were performed on the overlapping targets using the DAVID database (http://david.nifcrf.gov/). Official gene symbols were selected as the identifier, the gene list type was chosen, and the species was set to Homo sapiens. Significantly enriched pathways were screened with a threshold of *P* < 0.01. Visualization of GO enrichment results was performed using R 4.4.2 packages.

### Statistical analysis of data

All data are expressed as mean ± SEM. Results were statistically analyzed using GraphPad Prism version 8.0. Comparisons between the two groups were determined by Student t-test. For multiple comparisons, one-way ANOVA was used, followed by Tukey’s test. *P* < 0.05 was statistically significant.

## Results

### STDP ameliorates LCWE-induced vasculitis

**Fig. 1:**
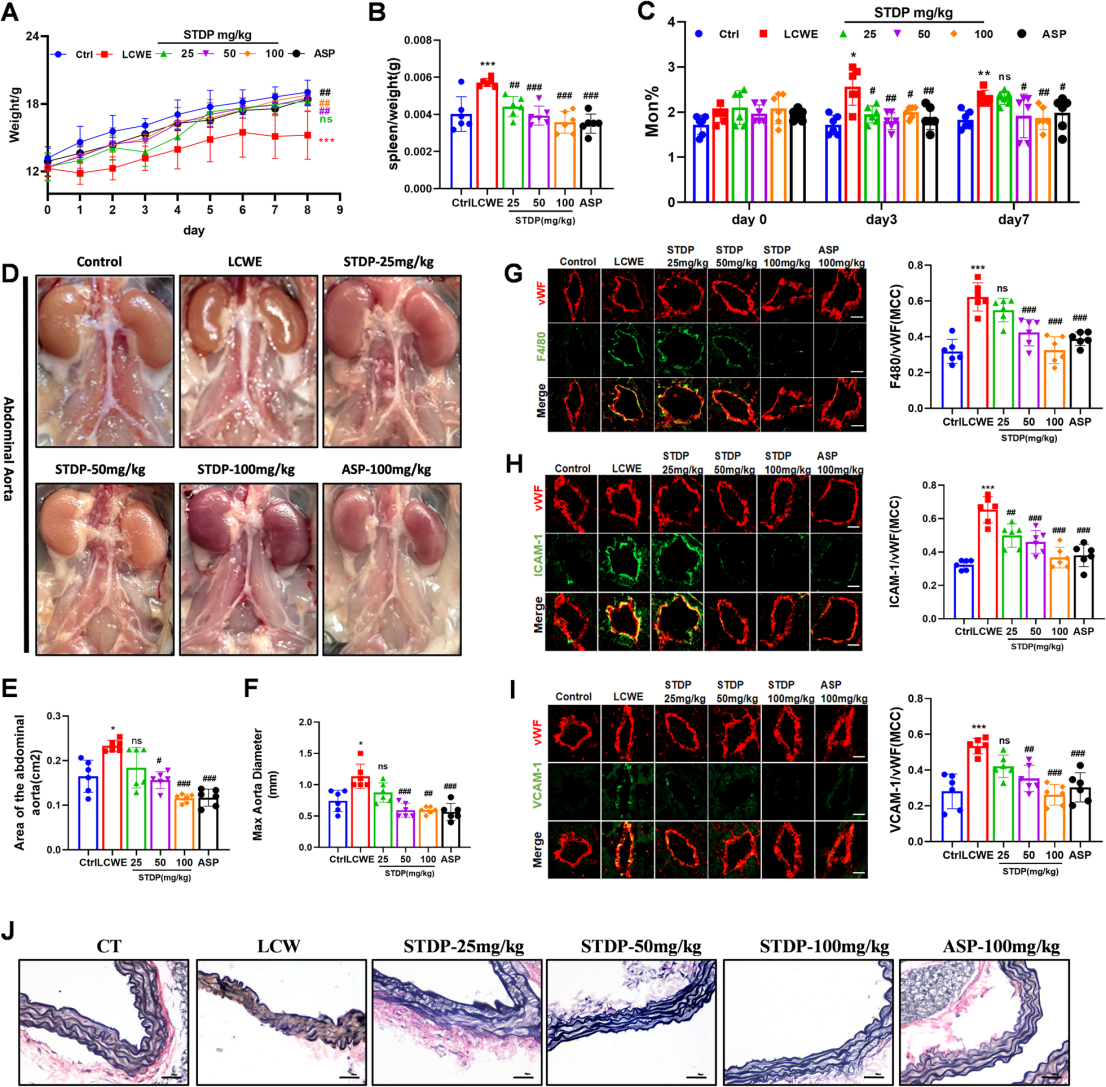
STDP ameliorates LCWE-induced vasculitis. **A** The weight changes of mice in each group. **B** Spleen coefficients of mice in each group. **C** Percentage of peripheral blood monocytes of mice in each group on days 0, 3, and 7. **D** Representative image about the abdominal aorta of mice in each group. **E** Statistical analysis about the abdominal aortic area of mice in each group. **F** Statistical analysis about the maximum diameter of the abdominal aorta of mice in each group. **G** Frozen sections of mouse hearts were taken for dual fluorescence co-localisation of vascular endothelial cells labelled with vWF (red) with F4/80 (green), scale bar = 10 μm. Statistical analysis of the Mandel co-localization coefficient between the two was performed. **H** Dual fluorescence co-localization detection was performed for vascular endothelial cells (labeled with vWF, red) and ICAM-1 (green), and statistical analysis was conducted using the Mandel co-localization coefficient, scale bar = 10 μm. **I** Dual fluorescence co-localization detection was performed for vascular endothelial cells (labeled with vWF, red) and VCAM-1 (green), and statistical analysis was conducted using the Mandel co-localization coefficient, scale bar = 10 μm. **J** Representative images of EVG staining of the mouse abdominal aorta in each group, scale bar = 50 μm. N = 6, *P < 0.05, **P < 0.01 compared to control group; ns *P* > 0.05, ^#^*P* < 0.05, ^##^*P* < 0.01, ^###^*P* < 0.001 compared to LCWE group

In order to evaluate the effect of STDP on LCWE-induced vasculitis, a mouse vasculitis model was established by intraperitoneal injection of LCWE. As reported, the pathogenesis of LCWE-induced vasculitis first occurs in the luminal endothelium and is characterised by a massive inflammatory cell infiltrate [32]. Injured endothelial cells express intercellular adhesion molecules and vascular cell adhesion molecules [9] leading to adhesion and chemotaxis of leukocytes such as monocytes [33]. The weight changes of mice in each group was detected during the experiment process, finding that STDP intervention could improve the weight loss induced by LCWE (Fig. 1A). And the spleen coefficient of mice in STDP groups decreased when compared with the model group (Fig. 1B). In addition, on the 3rd and 7th days, the proportion of monocytes in the blood of mice of STDP group decreased when compared with the model group (Fig. 1C). We investigated abdominal aortic dilation and the therapeutic amelioration of STDP in a LCWE-induced mouse model of vasculitis. Following standardized in situ perfusion-fixation under physiological pressure, accurate ex vivo morphometric analysis was performed to quantify the abdominal aortic area. Our results demonstrated that both the aortic luminal area and maximum diameter were significantly reduced in the medium- and high-dose STDP groups (Fig. 1D–F). To investigate the protective effect of STDP on the vasculature during vasculitis onset, we employed von Willebrand Factor (vWF) to locate endothelial cells co-localized with ICAM-1, VCAM-1, and F4/80. As shown in Fig. 1G–I, STDP administration significantly inhibited endothelial cell adhesion factor expression as well as reduced leukocyte adhesion and chemotaxis to endothelial cells when compared with model group. To evaluate the protective effect of STDP on vascular structural integrity, we performed EVG staining on mouse abdominal aortic sections. In the control group, the aortic wall exhibited well-organized elastic lamellae with continuous and parallel arrangement. In contrast, LCWE stimulation induced marked disruption of the elastic fiber architecture, characterized by fragmentation, disorganization, and loosening of the elastic lamellae. Notably, STDP treatment substantially ameliorated these pathological changes, preserving the layered structure and integrity of the elastic fibers (Fig. 1J). These findings demonstrate that STDP attenuates LCWE-induced vascular injury and maintains elastic fiber organization in the abdominal aorta.

### STDP attenuates LCWE-induced inflammation in MVECs

**Fig. 2:**
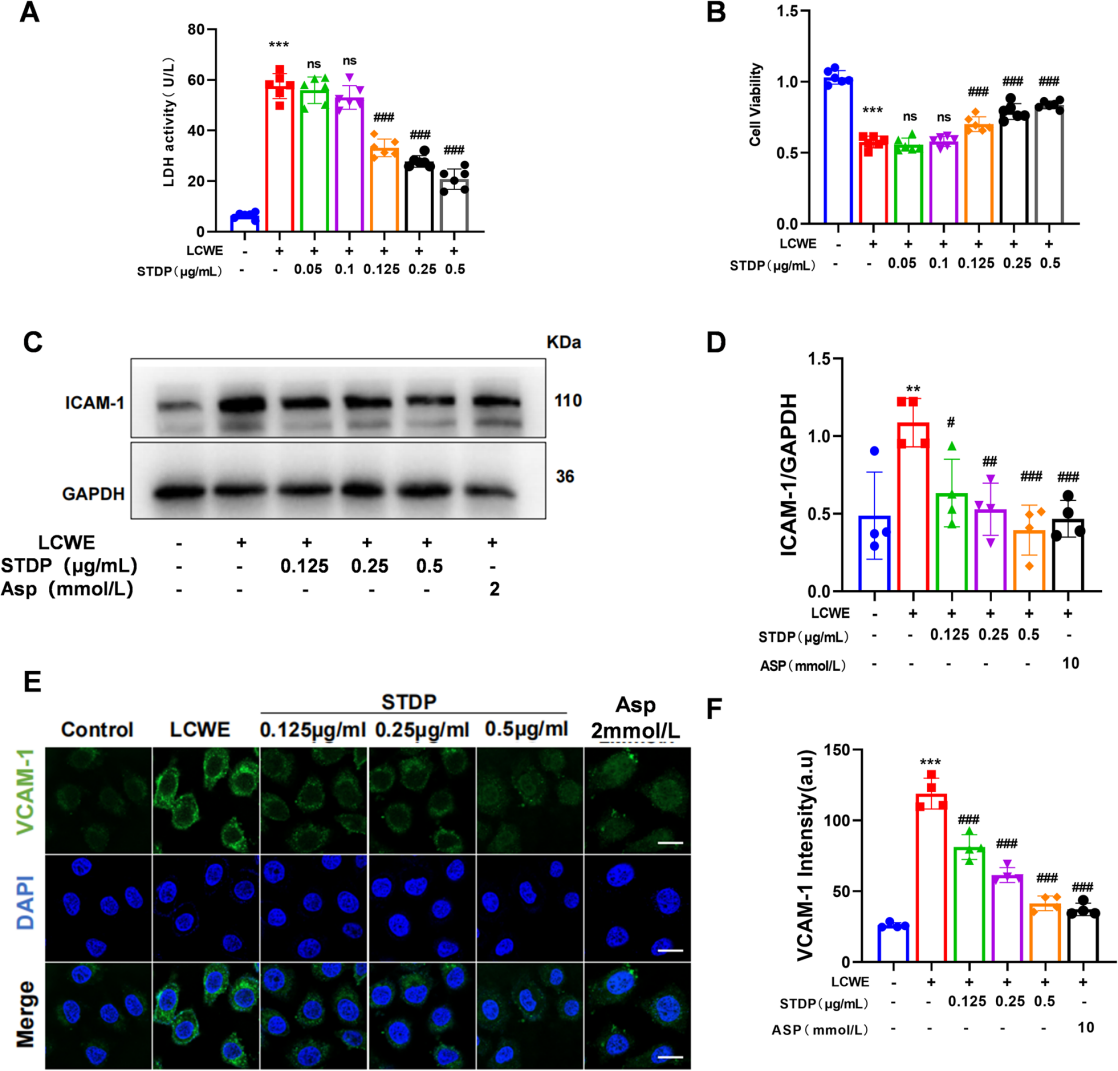
STDP attenuates the inflammation in LCWE-induced MVECs. **A** Cytotoxicity was assessed by LDH release assay. **B** Cell viability was measured by CCK-8 assay. **C, D** The expression level of ICAM-1 was analyzed by western blotting and the relative protein expression level were determined by densitometric analysis. **E, F** Representative fluorescent confocal images of VCAM-1 (green) and DAPI (blue) in MVECs and the summarized data of the immunofluorescence intensity, scale bar = 10 μm. N = 4–6, **P < 0.01, ***P < 0.001 compared to control group; ns *P* > 0.05, ^#^*P* < 0.05, ^##^*P* < 0.01, ^###^*P* < 0.001 compared to LCWE group

To further validate the protective effects of STDP against LCWE-induced inflammation, an in vitro model was established using MVECs stimulated with LCWE. The potential cytotoxic effects of STDP were initially assessed via CCK8 and LDH assays (Fig. 2A, B) to obtain non-cytotoxic concentrations for subsequent experiments, with reference to the dosing regimen previously reported by us [27]. Following treatment with STDP at the selected concentrations, the results showed that STDP administration reduced the expression of ICAM-1 and VCAM-1 on endothelial cells (Fig. 2C–F), which were consistent with those of animal experiments.

### STDP attenuates vascular endothelial dysfunction by inhibiting NLRP3 inflammasome activation

**Fig. 3:**
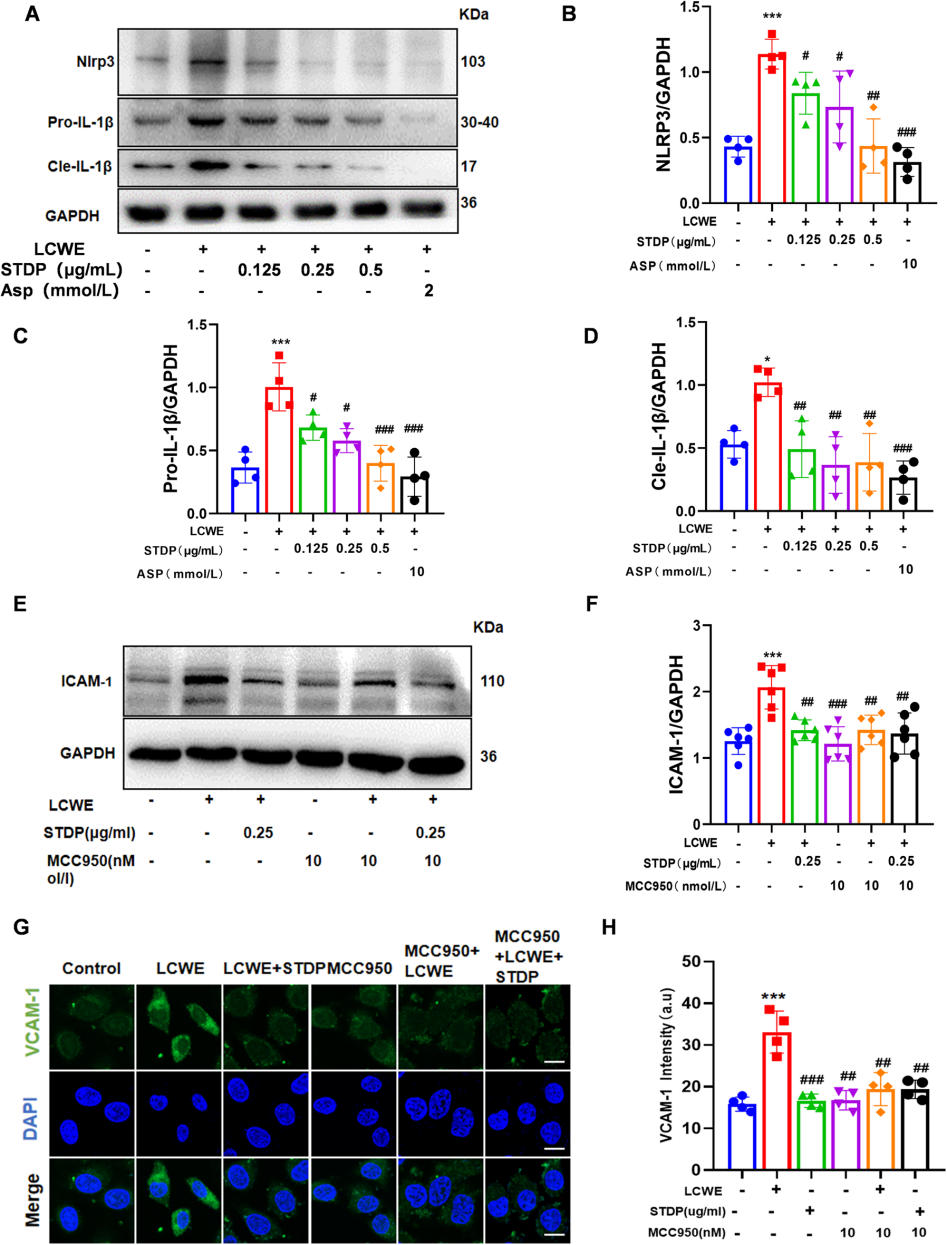
STDP attenuates vascular endothelial dysfunction by NLRP3 inhibition. **A**–**D** The expression levels of NLRP3, pro-IL-1β and cle-IL-1β were analyzed by western blotting and the relative protein expression level were determined by densitometric analysis. **E, F** The expression levels of ICMA-1 were analyzed by western blotting and the relative protein expression level were determined by densitometric analysis. **G, H** Representative fluorescent confocal images of VCAM-1 (green) and DAPI (blue) in MVECs and the summarized data of the immunofluorescence intensity, scale bar = 10 μm. N = 4–6, *P < 0.05, ***P < 0.001 compared to control group, ^#^*P* < 0.05, ^##^*P* < 0.01, ^###^*P* < 0.001 compared to LCWE group

It has been reported that the process of NLRP3 initiation and activation is closely related to endothelial damage and represents a potential target in inflammatory vascular conditions [34]. Additionally, the activation of IL-1β, which occurs after its initial activation, can target endothelial cells and stimulate the secretion of adhesion and chemokines by these cells [35]. To further investigate whether STDP ameliorates vascular endothelial dysfunction by suppressing NLRP3 inflammasome activation, the protein expression levels of NLRP3 and IL-1β was detected by western blotting, finding that STDP could significantly reduce the expression levels of NLRP3 and IL-1β (Fig. 3A–D). Furthermore, treatment with the NLRP3-specific inhibitor MCC950 markedly decreased the expression of ICAM-1 and VCAM-1 (Fig. 3E–H). Notably, when NLRP3 was inhibited by MCC950, additional administration of STDP did not induce any further significant reduction in ICAM-1 and VCAM-1 levels compared with the MCC950 + LCWE group. The absence of an additive effect when STDP was combined with MCC950 suggests that STDP acts upstream of or directly on the NLRP3 inflammasome pathway.

### STDP decreases Piezo1 expression in LCWE-induced MVECs 

**Fig. 4:**
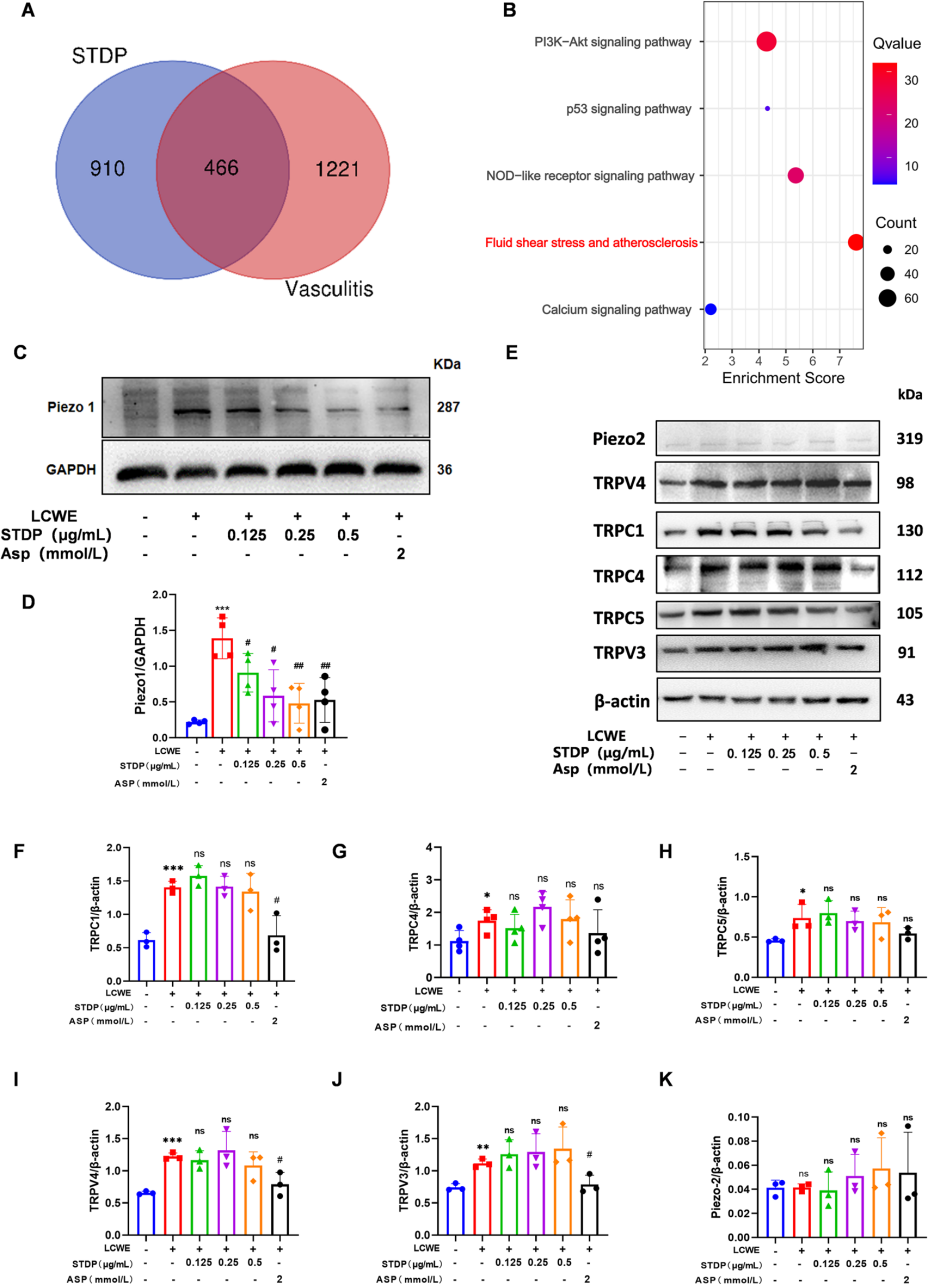
STDP decreases Piezo1 expression in LCWE-induced MVECs. **A, B** Among the key pathways identified through enrichment analysis of these targets, the fluid shear stress and atherosclerosis pathway showed the highest enrichment score. **C** LCWE stimulated MVECs and administered STDP as well as the positive drug ASP (2 mmol/L) Protein levels of Piezo1 were detected by WB. **D** Piezo1/GAPDH gray values were calculated for each group. **E**-**K** The expression levels of TRPC1, TRPC4, TRPC5, TRPV4, TRPV3 and Piezo2 were determined by Western blotting and the relative protein expression level were determined by densitometric analysis. N = 3–4, *P < 0.05, **P < 0.01, ***P < 0.001 compared to control group; ns *P* > 0.05, ^#^*P* < 0.05, ^##^*P* < 0.01 compared to LCWE group

It is becoming increasingly clear with multiple clinical evidence and in vivo experimental subjects that mechanical forces play a very important role in cardiovascular disease [36, 37]. When vascular inflammation occurs, disturbed blood flow shear, especially low and oscillatory flow in branching and curved vessel regions, leads to an inflammatory response in the endothelium. This response creates a distinct phenotype and promotes preferential sites of susceptibility to atherosclerosis [38]. Through network pharmacological analysis, a total of 466 intersection targets of STDP and vasculitis was discovered and the GO enrichment result showed that these intersection targets was enriched in “fluid shear stress and atherosclerosis” pathway (Fig. 4A, B).

To identify the key mechanosensitive molecule mediating this process, we examined the expression of several shear stress‑related proteins in response to LCWE stimulation and STDP treatment (Fig. 4E–K). Among these, Piezo1 is widely present in the cardiovascular system, and is a cation channel that is sensitive to blood flow shear, and whose opening manifests itself as an event that causes the concentration of Ca^2+^ in the cell to elevation [38], and one of the activation pathways of NLRP3 is increased Ca^2+^ inward flow. LCWE stimulation significantly upregulated Piezo1 protein expression, whereas other shear‑responsive proteins exhibited only minor or no significant changes. And the results of western blot analysis revealed that LCWE stimulation significantly upregulated Piezo1 protein expression, whereas STDP intervention markedly attenuated this effect (Fig. 4C, D).

### STDP attenuates inflammasome activation via regulation of Ca^2+^ signaling

**Fig. 5:**
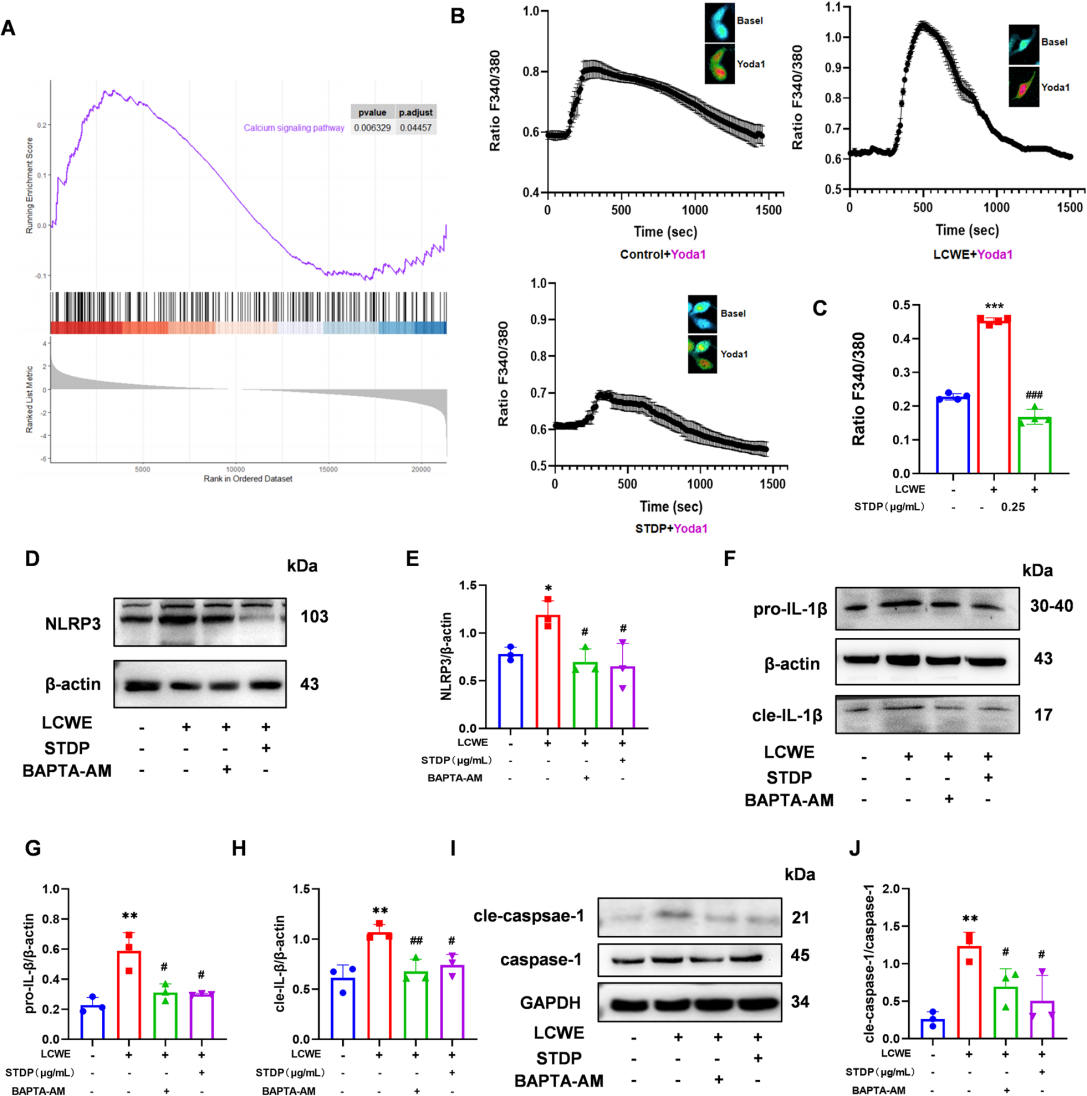
STDP attenuates inflammasome activation via regulation of Ca^2+^ signaling. **A** In the differential analysis of gene expression matrices in vasculitis diseases, the Ca^2+^ pathway was significantly enriched. **B, C** MVECs in each group were observed under the Ca^2+^ imaging equipment and 5 mmol/L of Yoda1 (Piezo1 channel agonist) was added to cells after the baseline was smooth for about 1500 s, and the average of peak and baseline Ca^2+^ concentration was calculated in eight cells selected from each group. Representative images and statistical charts of intracellular Ca^2+^ changes. **D–J** The protein expression levels of NLRP3, cleaved caspase-1 (cle-caspase1), and IL-1β were analyzed by Western blotting and the relative protein expression level were determined by densitometric analysis. N = 3–4, *P < 0.05, **P < 0.01, ***P < 0.001 compared to control group, ^#^*P* < 0.05, ^##^*P* < 0.01, ^###^*P* < 0.001 compared to LCWE group

Bioinformatics analysis revealed a significant enrichment of Ca^2+^ signaling pathways in vasculitis (Fig. 5A), suggesting that dysregulation of Ca^2+^ homeostasis may contribute to the disease pathogenesis. To investigate whether calcium influx is causally involved in the inflammatory response, we first examined the effect of STDP on Piezo1-mediated Ca^2+^ influx. As shown in Fig. 5B, C, STDP markedly inhibited Piezo1 channel activation in MVECs and reduced Ca^2+^ influx. Given that Ca^2^⁺ acts as key second messenger that can trigger inflammasome activation, we hypothesized that STDP may suppress inflammation by limiting Ca^2+^-dependent signaling. To prove this hypothesis, we employed a Ca^2+^ chelator (BAPTA-AM) to directly sequester intracellular Ca^2+^ and compared its effects with those of STDP on downstream inflammatory mediators. LCWE stimulation robustly upregulated NLRP3, IL-1β and Caspase-1 expression, indicating inflammasome activation. Notably, both the Ca^2+^ chelator and STDP significantly attenuated these increases, and the magnitude of inhibition was comparable between the two treatments (Fig. 5 D-J). These results demonstrate that Ca^2^⁺ signaling is an essential upstream event in LCWE-induced inflammasome activation, and that STDP exerts its anti-inflammatory effects primarily through suppressing Ca^2^⁺ influx via Piezo1 channels.

### Piezo1 knockdown abrogates STDP-mediated endothelial protection

**Fig. 6:**
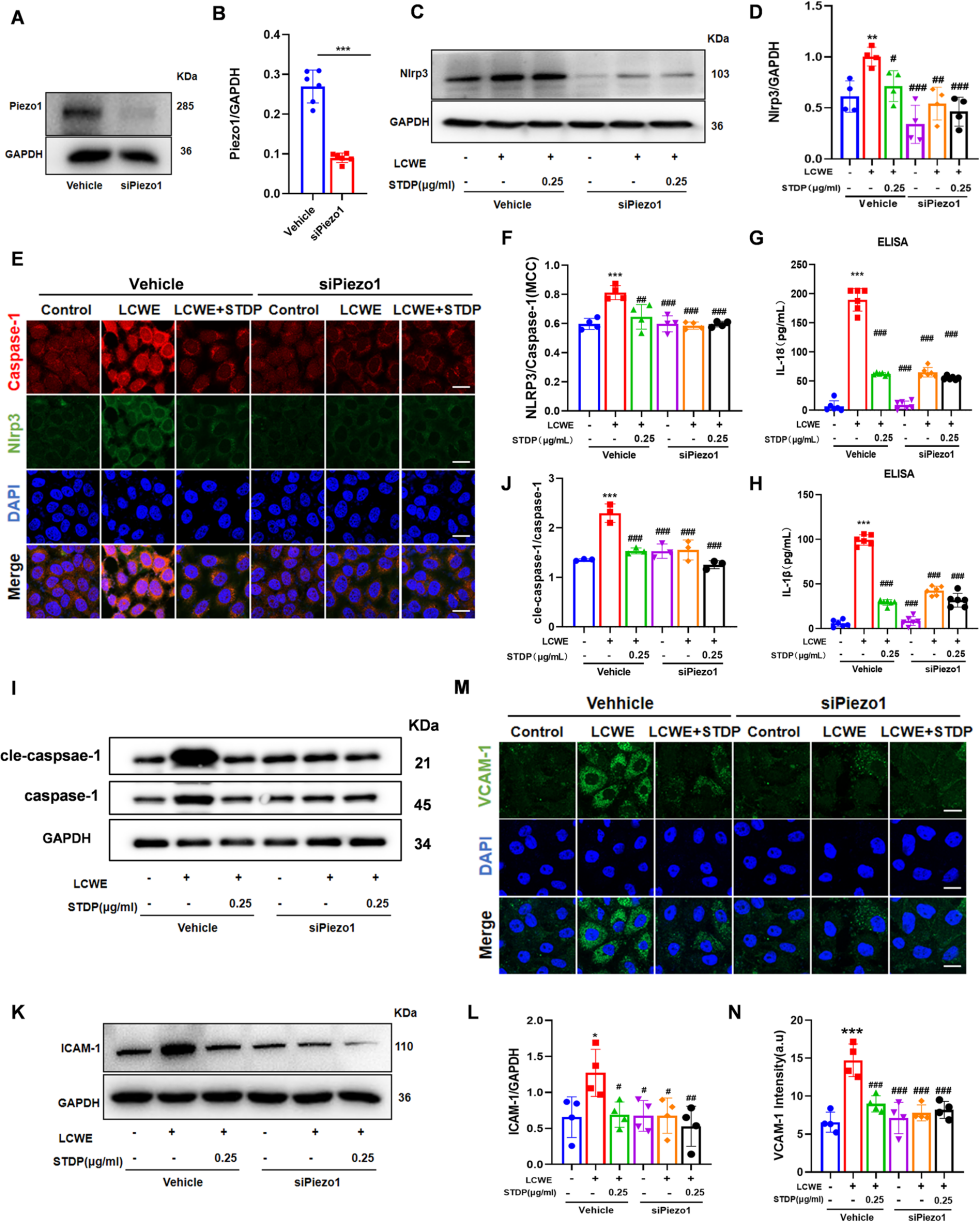
Piezo1 knockdown abrogates STDP-mediated endothelial protection. **A, B** Knockdown Piezo1 representative images as well as statistical graphs. **C, D** The protein expression levels of NLRP3 was analyzed by Western blotting and the relative protein expression level were determined by densitometric analysis. **E, F** Immunofluorescence double staining to co-localize NLRP3 with Caspase1. **G, H** The levels of IL-18 and IL-1β in cell culture supernatants were measured by ELISA. **I, J** The expression levels of pro-Caspase-1, cle-Caspase-1 were analyzed by Western blotting and the relative protein expression level were determined by densitometric analysis. **K, L** The protein expression levels of ICAM-1 was analyzed by Western blotting and the relative protein expression level were determined by densitometric analysis. **M, N** Representative fluorescent confocal images of VCAM-1 (green) and DAPI (blue) in MVECs and the summarized data of the immunofluorescence intensity, scale bar = 10 μm. N = 4–6, *P < 0.05, **P < 0.01, ***P < 0.001 compared to control group, ^#^*P* < 0.05, ^##^*P* < 0.01, ^###^*P* < 0.001 compared to LCWE group

To determine whether STDP-mediated endothelial protection requires Piezo1-dependent Ca^2+^/NLRP3 signaling, we performed Piezo1 knockdown using siRNA (Fig. 6A, B). After 24 h post-transfection, endothelial cells were treated with LCWE or LCWE + STDP in both Piezo1-knockdown and control groups. Notably, Piezo1 silencing abolished LCWE-induced NLRP3 upregulation (Fig. 6C, D) and activation of the downstream inflammasome pathway, as demonstrated by reduced caspase-1 cleavage (Fig. 6 I–J) and diminished secretion of IL-1β and IL-18 (Fig. 6 G-H). Consistently, immunofluorescence co-localization of NLRP3 and Caspase-1 was also attenuated by Piezo1 knockdown (Fig. 6E–F). These changes were accompanied by concomitant reductions in ICAM-1 and VCAM-1 expression (Fig. 6K–N). Crucially, STDP failed to exert further inhibitory effects on these inflammatory markers in Piezo1-deficient cells, indicating that its anti-inflammatory action is dependent on Piezo1.

## Discussion

Vascular endothelial inflammation is a central pathological process underlying various inflammatory vascular conditions. The mechanosensitive ion channel Piezo1 has recently emerged as a key driver of endothelial inflammation and vascular remodeling [39]. Given that certain bioactive components of Shexiang Tongxin Dropping Pills (STDP) have been shown to modulate Ca^2+^ signaling and ion channel activity [40], we hypothesized that STDP might exert its vascular protective effects by targeting this mechanosensitive axis. In this study, we demonstrate that STDP attenuates LCWE-induced vascular inflammation by targeting the Piezo1/Ca^2+^/NLRP3 signaling axis, thereby uncovering a previously unrecognized mechanism of STDP and highlighting the critical role of mechanotransduction in driving endothelial inflammation.

The protective effects of STDP on vascular structure were evidenced by the attenuation of abdominal aortic dilation and preservation of elastic fiber integrity (Fig. 1D–F, J). These morphological improvements are likely attributed to the inhibition of endothelial activation, as STDP suppressed LCWE-induced upregulation of ICAM-1 and VCAM-1 (Fig. 1H–I, Fig. 2C–F), thereby reducing leukocyte adhesion (Fig. 1G). This aligns with the established role of STDP in cerebral microvascular protection [27] and extends its relevance to the initial and precipitating events of vascular inflammation, such as endothelial injury and subsequent leukocyte recruitment.

A key finding of our study is the identification of the Piezo1 channel as a critical upstream mediator of endothelial inflammation in this model of vascular inflammation. Although Piezo1 is known to sense hemodynamic forces, its pathological upregulation in response to inflammatory stimuli (LCWE) has not been previously reported. Our systematic screening of mechanosensitive channels confirmed the specificity of Piezo1 in this context, as only Piezo1 exhibited significant LCWE-induced upregulation that was reversed by STDP (Fig. 4C, D).

Mechanistically, we demonstrate that STDP disrupts the Piezo1/Ca^2^^+^/NLRP3 cascade. LCWE-induced Ca^2+^ influx was dependent on Piezo1, as genetic silencing of Piezo1 abolished calcium mobilization and subsequent NLRP3 inflammasome activation. The observation that STDP failed to exert additional inhibitory effects in Piezo1-deficient cells (Fig. 6E–N) strongly suggests that Piezo1 is a necessary node for STDP’s anti-inflammatory action. Furthermore, the pharmacological chelation of Ca^2+^ with BAPTA-AM phenocopied the effects of STDP on the NLRP3 inflammasome (Fig. 5D–J), reinforcing the centrality of calcium signaling in this pathway. These findings position STDP as a modulator of endothelial calcium homeostasis, a mechanism that distinguishes it from conventional anti-inflammatory agents.

Recent work by Wang et al.[41] demonstrated that quercetin inhibits Piezo1 channel activity in atherosclerosis. Our study both complements and extends these findings. First, we establish the pathogenic relevance of Piezo1 in an acute experimental vasculitis model, broadening its implication beyond chronic atherosclerotic disease. Moreover, unlike single-compound agents such as quercetin, STDP is a multicomponent traditional Chinese medicine formulation with established clinical safety. Our findings identify a previously unrecognized mechanism of STDP that involves targeting Piezo1 expression, which may underlie its effects in this model. Furthermore, the comparable inhibitory effects of STDP and the calcium chelator BAPTA-AM on NLRP3 inflammasome activation (Fig. 5D–J) provide direct pharmacological evidence that calcium signaling serves as a critical upstream event in this inflammatory process. This observation aligns with the emerging concept that modulation of calcium homeostasis may confer therapeutic benefits in inflammatory diseases, and positions STDP as a promising candidate for such intervention. Genetic evidence from siRNA-mediated Piezo1 knockdown provides the strongest support for our conclusions.

The core innovation of this study lies in its drug repurposing strategy, exploring STDP’s therapeutic potential in vascular inflammation beyond its traditional indication for adult ischemic heart disease. By linking STDP’s anti-inflammatory and endothelial protective properties to the core pathological mechanisms of vascular endothelial inflammation, we complement the mechanistic exploration of applying STDP to vascular injury and provide preliminary evidence for its potential application in inflammatory vascular conditions.

However, several limitations should be acknowledged in this study. First of all, while STDP has established safety in adults [42], its safety and pharmacokinetics in other populations remain unknown. In addition, our study focused on acute inflammatory responses; long-term effects on vascular remodeling and aneurysm formation remain to be determined. Besides, although the LCWE-induced mouse model recapitulates key pathological features of vascular inflammation, it cannot fully replicate all clinical manifestations of human inflammatory vascular disease. Therefore, further validation in other experimental models and translational studies is necessary.

## Conclusion

**Fig. 7:**
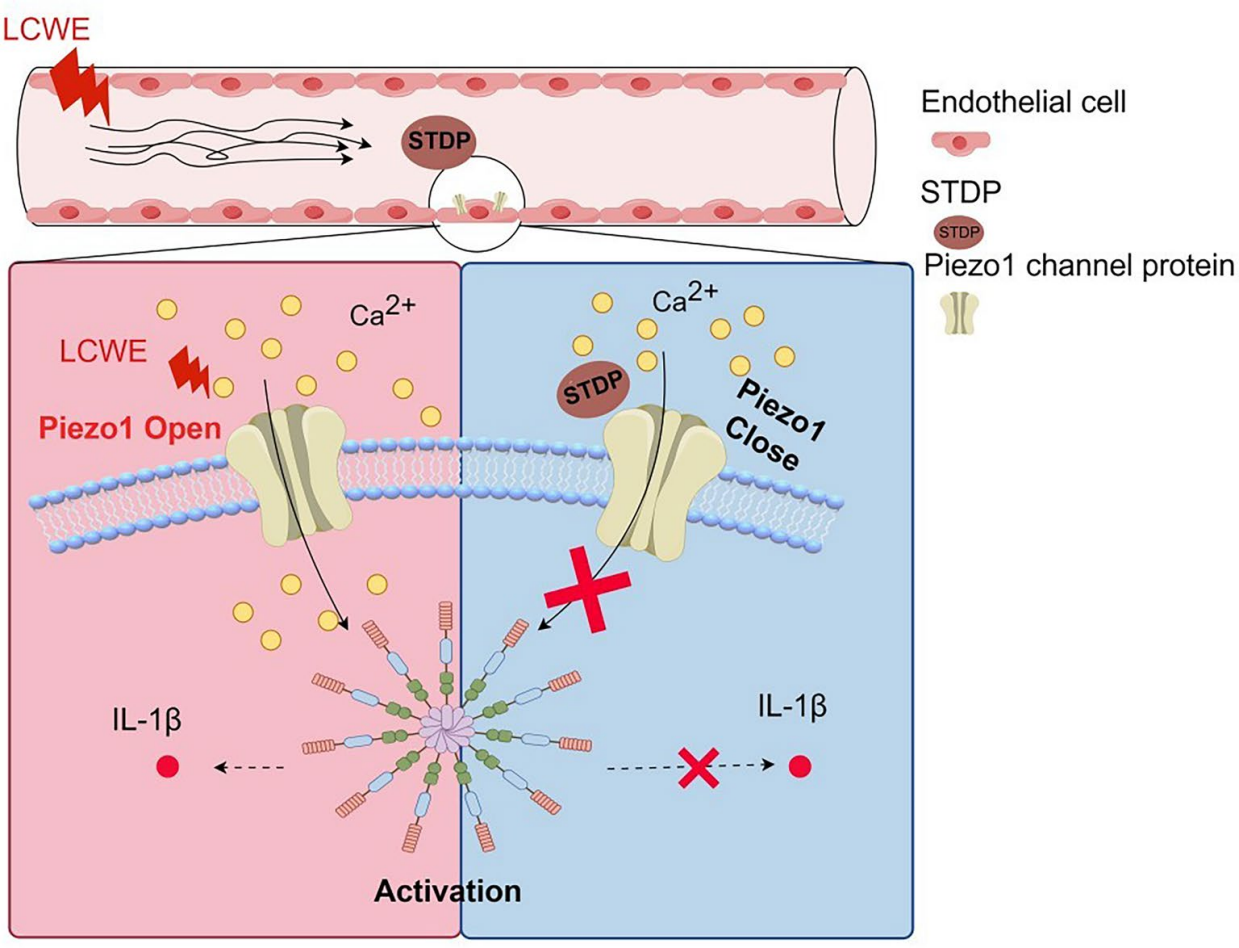
STDP attenuates vascular endothelial inflammation by modulating the Piezo1/Ca^2+^/NLRP3 signaling axis

This study demonstrates that STDP protects endothelial cells against LCWE-induced vascular endothelial inflammation in both in vitro and in vivo models. Mechanistically, STDP attenuates vascular endothelial inflammation by modulating the Piezo1/Ca^2+^/NLRP3 signaling axis (Fig. 7). These findings reveal novel pharmacological effects of STDP and advance our understanding of the molecular mechanisms underlying vascular endothelial inflammation.

In the LCWE-induced model of vascular inflammation, STDP therapy exerts its effects by inhibiting Piezo1, a mechanosensitive ion channel in vascular endothelial cells. This inhibition reduces calcium ion (Ca^2+^) influx, which in turn suppresses the activation of the NLRP3 inflammasome. The attenuation of endothelial inflammation is manifested by the downregulation of adhesion molecules (ICAM-1, VCAM-1) and reduced macrophage infiltration, ultimately protecting blood vessels from damage and preserving the integrity of arterial elastic fibers.

## Data Availability

The research data used to support the study are available from the corresponding author upon reasonable request.

## Abbreviations

STDP: Shexiang Tongxin Dropping Pills
LCWE: Lactobacillus casei cell wall extract
MVECs: Microvascular endothelial cells
Ca^2+^: Calcium ion
vWF: Von Willebrand Factor
VCAM-1: Vascular cell adhesion molecule-1
ICAM-1: Intercellular adhesion molecule-1
NLRP3: NOD-like receptor family, pyrin domain containing 3
Piezo1: Piezo—type mechanosensitive ion channel component 1
CALs: Coronary artery lesions
ATCC: American Type Culture Collection
